# Genetic alterations in lymphoblastic leukaemia** / lymphoma – a practical guide to WHO HAEM5**

**DOI:** 10.1515/medgen-2024-2007

**Published:** 2024-03-06

**Authors:** Doris Steinemann, Małgorzata Dawidowska, Lisa J Russell, Christine J Harrison, Gudrun Göhring

**Affiliations:** Hannover Medical School Department of Human Genetics Hannover Germany; Institute of Human Genetics Department of Molecular and Clinical Genetics Poznan Poland; Newcastle University Centre for Cancer Biosciences Institute Newcastle upon Tyne UK; Newcastle University Centre for Cancer Leukaemia Research Cytogenetics Group, Translational and Clinical Research Institute, Newcastle upon Tyne UK; Amedes genetics MVZ wagnerstibbe für Laboratoriumsmedizin, Hämostaseologie, Humangenetik und Mikrobiologie Hannover Germany

## Abstract

We present a practical guide for analyzing the genetic aspects of lymphoblastic leukaemia/lymphoma according to the 5th edition of the World Health Organization (WHO) classification of haematolymphoid neoplasms (WHO-HAEM5) issued in 2024. The WHO-HAEM5 acknowledges the increasing importance of genetics in the diagnosis of lymphoid neoplasia. Classification is based on the established genetic subtypes according to cell lineage, with precursor cell neoplasms followed by mature malignancies. This guide describes those genetic abnormalities in acute precursor B- and T-cell neoplasms required for risk stratification, and for treatment, providing diagnostic algorithms under the headings of ‘essential’ and ‘desirable’ diagnostic criteria.

Keywords: WHO HAEM5 classification, Precursor B-cell neoplasms, B-ALL, T-ALL, LBL

## Introduction

In the revised 5th edition of the World Health Organization (WHO) classification of haematolymphoid neoplasms (WHO-HAEM5), lymphoblastic leukaemia (ALL) and lymphoma (LBL) are grouped together into lymphoblastic neoplasms of B- and T-precursor cells [1]. The distinction between leukaemia and lymphoma depends on the primary involvement of blood and bone marrow (ALL) or lymph node and/or extra-nodal sites (LBL). The extensive development and integration of new genetic methods into routine diagnostics has greatly impacted the revised classification. While for the B-cell ALL/LBL, new genetic subgroups have been defined, in T-ALL/LBL, the classification remains based on immunophenotyping and histology.

## B-ALL and lymphoma

ALL is the most frequent cancer in childhood, with B-ALL accounting for around 80 % of cases. The annual incidence rate of ALL is 3–4 cases per 100,000 children and young adults. The peak incidence occurs in young children [2], with long-term cure achieved in nearly 90 % of cases on most international contemporary protocols [3–6]. Adult ALL is rare and has inferior outcomes compared to children. Good-risk subtypes are more common in paediatric cases, while adverse-risk subtypes are more prevalent in adults [7]. Treatment is based on risk of relapse, predicted from a combination of clinical (e. g., age, white blood cell count), genetic, and morphological early response criteria. Monitoring of leukaemic blast clearance reflects treatment response and measurable residual disease (MRD), in association with genetics, are strong prognostic factors in ALL [4, 8]. B-LBL are morphologically identical to ALL, comprising around 10 % of LBL.

### B-ALL/LBL genetic subclassification

**Figure 1:**
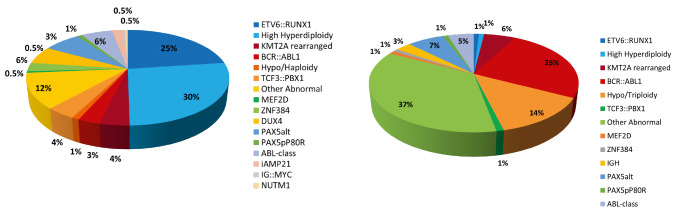
Frequency of B-ALL/LBL subtypes in children (left) and adults (right)

A broad spectrum of established genetic aberrations with prognostic impact are used in risk stratification of B-ALL/LBL [9, 10]. These genetic markers may be numerical (aneuploidies) or structural (translocations, copy number variants) and stratify patients into low, standard, high or very high-risk arms, differing in treatment intensity. High hyperdiploidy (HHD) is the largest genetic subgroup, present in approximately 30 % of paediatric but only 1 % of adult B-ALL/LBL (Fig. 1). It is defined by a non-random pattern of chromosomal gains (X, 4, 6, 10, 14, 17, 18, and 21) resulting in a modal chromosome number of 51–67 chromosomes [11]. The overall prognosis is excellent. In contrast, hypodiploidy, defined by a modal number of 43 chromosomes or less, has a poor outcome. B-ALL/LBL with intrachromosomal amplification of chromosome 21 – iAMP21-ALL – was first described in 2003 as a distinct entity comprising around 2 % of paediatric B-ALL, with a median age of 9 years [12]. This subtype is also associated with a poor prognosis and benefits from treatment intensification [13]. Other markers of poor prognosis are the gene fusions: *BCR::ABL1* resulting from the translocation t(9;22)(q34;q11), *TCF3::HLF1/*t(17;19)(q22;p13) and a range of *KMT2A* fusions including the most frequent partner genes: *AFF1*, *MLLT1*, *MLLT3*, *MLLT10*, *AFDN*, *EPS15* and *USP2* [14]. The rare fusion, *TCF3::HLF*, is a new entity within WHO-HAEM5. Whilst B-ALL/LBL harbouring this aberration is resistant to conventional chemotherapies, it has shown sensitivity to the BCL2-specific inhibitor venetoclax [15].

The *ETV6::RUNX1* fusion, resulting from the translocation, t(12;21)(p13;q22) is associated with favorable outcome [16, 17]. B-ALL with a balanced t(1;19)(q23;p13) or its unbalanced form, der(19)t(1;19), giving rise to *TCF3*::*PBX1* fusion, has an intermediate prognosis [18]. B-ALL/LBL with *IG*::*IL3* fusion/t(5;14)(q31.1;q32.1) or other cryptic insertional rearrangements is a rare entity associated with accumulation of eosinophils. Herein, the *IGH* super-enhancers (14q32.1) are juxtaposed to the vicinity of the *IL3* gene (5q31.1) leading to IL3 overexpression [19, 20].

B-ALL/LBL with *BCR*::*ABL1*-like features was introduced in WHO-HAEM4R, but *ETV6*::*RUNX1*-like-ALL is newly included in WHO-HAEM5. Although these entities lack the *BCR*::*ABL1* and* ETV6*::*RUNX1* fusions, respectively, they show similar gene expression profiles [21, 22]. *ETV6*::*RUNX1*-like ALL typically harbours deletions targeting *ETV6* and *IKZF1* [21].

### B-ALL/LBL with other defined genetic abnormalities

Around 30 % of adult and 15 % of childhood B-ALL/LBL do not have established genetic abnormalities at diagnosis [23]. These cases were previously defined as B-other-ALL with highly variable prognosis and treatment response. Recently, whole genome- and transcriptome-sequencing have identified multiple new genomic subtypes of B-ALL. However, evidence for defining them as potential novel entities conferring distinct clinical, phenotypic and/or prognostic effects is limited. Thus, these new subtypes are listed under “B-ALL/LBL with other defined genetic abnormalities”. They include B-ALL/LBL with *DUX4*, *MEF2D*, *ZNF384* or *NUTM1* rearrangements, *IG*::*MYC* fusion, and cases with *PAX5* alterations or *PAX5* p.P80R. B-ALL/LBL that do not show a recurrent genetic alteration after comprehensive testing are summarized under NOS (not otherwise specified).

**Table 1:** WHO Classification of B-lymphoblastic leukaemias/lymphomas (B-ALL/LBL) and of T-cell lymphoblastic leukaemia/lymphoma (T-ALL/LBL)From: The 5th edition of the World Health Organization Classification of Haematolymphoid Tumours: Lymphoid Neoplasms

**5^th^ edition of WHO classification**
**B-ALL/LBL**
B-lymphoblastic leukaemia/lymphoma, NOS
B-lymphoblastic leukaemia/lymphoma with **high hyperdiploidy (HHD)**
B-lymphoblastic leukaemia/lymphoma with **hypodiploidy**
B-lymphoblastic leukaemia/lymphoma with **iAMP21**
B-lymphoblastic leukaemia/lymphoma with ***BCR*::*ABL1* fusion**
B-lymphoblastic leukaemia/lymphoma with ***BCR*::*ABL1*-like features**
B-lymphoblastic leukaemia/lymphoma with ***KMT2A* rearrangement**
B-lymphoblastic leukaemia/lymphoma with ***ETV6*::*RUNX1* fusion**
B-lymphoblastic leukaemia/lymphoma with ***ETV6*::*RUNX1*-like features**
B-lymphoblastic leukaemia/lymphoma with ***TCF3::PBX1* fusion**
B-lymphoblastic leukaemia/lymphoma with ***IGH*::*IL3 fusion***
B-lymphoblastic leukaemia/lymphoma with ***TCF3*::*HLF* fusion**
B-lymphoblastic leukaemia/lymphoma with **other defined genetic abnormalities**
**T-ALL/LBL**
T-lymphoblastic leukaemia / lymphoma, NOS
Early T-precursor lymphoblastic leukaemia / lymphoma

NOS: not otherwise specified

***DUX4*-rearranged:** Insertions of *DUX4* close to the *IGH* super-enhancers and into intron 3 of the *ERG* locus have been described in about 4 % of B-ALL/LBL [21]. Frequent *ERG* deletions and a specific expression profile have been associated with this group [21, 24]. The detection of *DUX4* rearrangements is challenging due to the small size of the rearrangement, the macrosatellite repeat nature of the *DUX4* locus, as well as its subtelomeric localization. However, increased expression of *DUX4* from RNA sequencing, immunohistochemical staining of CD2 [25], and CD371 expression by flow cytometry [26] facilitate identification of these cases. B-ALL/LBL with *DUX4* rearrangements is associated with low relapse rates and high overall survival, despite persistent MRD early in the treatment course [27].
***MEF2D*-rearranged:** *MEF2D*::*BCL9* and *MEF2D*::*HNRNPUL1* are the most common fusions, although other fusion partners have been rarely reported, including *FOXJ2, CSF1R, HNRNPH1, PYGO2, BCL9L* and* SS18* [28]. A high risk of relapse has been associated with *MEF2D*::*BCL9* fusions, but is not seen in patients with other *MEF2D* partners [28].
***ZNF384*-rearranged:** Around 20 *ZNF384* fusion partners have been identified so far, including *EP300*, *TCF3*, *TAF15*, *CREBBP*,* EWSR1*. The clinical significance of each fusion partner remains unclear due to the small number of reported cases. However, patients with *EP300*::*ZNF384* ALL have been shown to have a lower cumulative relapse rate than other fusions [29].
***NUTM1*-rearranged:** B-ALL/LBL with *NUTM1* rearrangements, although rare, occur more frequently in infants without *KMT2A* rearrangements. They appear to have a favorable outcome [30].
***IG*::*MYC*:** Although *IG*::*MYC* translocations are typical of Burkitt lymphoma (BL), WHO-HAEM5 reports infrequent cases of BL with a phenotype of precursor B-cells including expression of terminal deoxynucleotidyl transferase, sometimes CD34, and absence of CD20 and surface immunoglobulin expression. Not only their immunophenotype but also their molecular profiles, with frequent RAS-pathway mutations, and a distinct methylome indicate B-ALL/LBL rather than BL, where the *IG*::*MYC* translocation occurs at an early stage of B-cell maturation [31].
***PAX5* alterations:** Monoallelic deletions, ranging from focal to whole chromosome 9 deletions, are the most frequent *PAX5* alterations in B-ALL/LBL. They act as cooperating events requiring other oncogenic lesions to induce overt malignant transformation. In contrast, *PAX5* fusions – of which *PAX5*::*ETV6* is the most recurrent – are founder lesions, displaying a relatively simple karyotype. *PAX5* intragenic amplification (*PAX5*amp) has also been included in this subgroup based on similar RNA expression profiles [32].
***PAX5* p.P80R:** This subtype is characterized by biallelic alterations of *PAX5,* whereby one *PAX5* allele acquires a deleterious mutation together with deletion or copy-neutral loss of heterozygosity of the WT allele. A reduced overall survival has been associated with this subtype [33].

### T-cell acute lymphoblastic leukaemia and lymphoma (T-ALL/LBL)

The classification of T-ALL/LBL, broadly used in the literature, is complex and based on the oncogenic activation of several transcription factors (TF), driving leukaemia development and defining major genetic subtypes: *TAL1*, *TAL2, TLX1, TLX3*, *HOXA*, *LMO1/2*, *LMO2/LYL1*, *NKX2-1*, *SPI1* [34, 35]. Physiologically, these TF are involved in the development and maturation of T-cell precursors. In T-ALL/LBL they show ectopic expression due to rearrangements with TCR gene enhancers, structural variants or smaller mutations creating novel binding sites for factors enhancing the expression of these TF. Regardless of the mechanism, their oncogenic activation leads to a maturation arrest of T-cell precursors and to subtype-specific transcriptional reprograming. These genetic subtypes have been correlated with the maturation stages and with the presence of some genetic aberrations, e. g. *TLX1* activation is frequently associated with a cortical immunophenotype; *LMO2/LYL1* and *HOXA* subtypes are prevalent in ETP-ALL/LBL and are frequently associated with mutations activating JAK-STAT signaling; *NUP214*::*ABL* fusions are more prevalent in the *TLX1/TLX3* subtype [36, 37] (Sin et al. 2021).

These proposed subtypes were defined by RNA-sequencing / gene expression profiles / RT-qPCR (to detect ectopic expression of TF) or genomic approaches (to detect the underlying genetic aberration, e. g. *TCR*::*TF* fusion). Increasing data from high throughput approaches, RNA-sequencing in particular, have shown that distinction between genetic subtypes is not clear-cut (some cases show expression of more than one TF driver oncogene). Despite improved understanding of the genetic landscape of T-ALL/LBL, evidence for the clinical relevance of the proposed genetic subtypes is lacking. WHO-HAEM5 includes only two entities: T-lymphoblastic leukaemia/lymphoma, NOS and Early T-precursor lymphoblastic leukaemia/lymphoma (ETP-ALL/LBL). The latter entity comprises approx. 5–17 % of paediatric and 7 % of adult T-ALL cases [37]. ETP-ALL/LBL is identified by similarity to earlier stages of T-cell precursors, based on gene expression profiling and detection of stem cell/myeloid markers by flow cytometry. The NK-lymphoblastic leukaemia/lymphoma was defined as a provisional entity in the WHO-HAEM4R, but it is no longer included in WHO-HAEM5 due to insufficient credibility of a distinct entity and the diagnostic criteria.

### Diagnostic techniques for the detection of numerical and structural variants

A range of methods are currently used for the detection of genetic abnormalities in ALL/LBL. Most of the established abnormalities, defining the important genetic subtypes, can be recognized by cytogenetic testing. Karyotyping and fluorescence *in-situ* hybridization (FISH) have been used to identify good-risk (e. g. high hyperdiploidy and *ETV6::RUNX1* fusion) and poor-risk (*KMT2A* rearrangements, *BCR::ABL1,* and hypodiploidy) subtypes of childhood B-ALL/LBL [38]. However, there is no clear agreement on the optimal method to define high hyperdiploidy. Karyotyping and/or DNA index (DI), a measure of the overall DNA content, were traditionally used. However, other genomic testing methods that allow identification of specific trisomies (e. g. WGS, SNP arrays, RNA-sequencing, FISH) are also being used. As increasing numbers of new and rare subtypes are being identified, it is becoming impossible to identify them all by karyotyping and FISH due to their limitations of resolution and time spent in analysis. For example, detection of *ZNF384* and its fusion partners is challenging because they both are located close to telomeres. Identification of *DUX4* rearrangements are impossible due to the small size of the insertions as well as the repetitive nature of its sequence, also challenging its detection by WGS. *TCF3*::*HLF* can be cryptic, thus RNA sequencing may be the best technique for its detection [39]. Optical genome mapping (OGM) has recently been introduced as an all-in-one high-resolution cytogenetic technique that is able to detect balanced and unbalanced translocations, the full range of copy number variations from a few kilobases to the chromosome level (aneuploidies), as well as genomic insertions and inversions [40]. However, it is unable to detect single nucleotide variants. High specificity and sensitivity of the chosen method(s) are important, as low tumour cell counts may further compromise detection.

### Immunophenotyping

Immunophenotype characterization by flow cytometry is an essential tool for ALL/LBL diagnostics. WHO-HAEM5 continues to subclassify B-ALL/LBL and T-ALL/LBL by immunophenotype and no changes to flow cytometry criteria have been made. B-ALL/LBL typically expresses CD10, CD19, CD22, TdT, CD34, HLA-DR and CD45 (normal, diminished or negative) antigens, and is negative for surface immunoglobulin. Some cases have a more differentiated immunophenotype with slightly increased CD45 and diminished CD34 intensity, while expressing cytoplasmic immunoglobulin heavy chains (cµ). Interestingly, a correlation between phenotypic features and genetic subtypes has been observed. B-ALL/LBL with *ETV6*::*RUNX1* shows intense expression of CD10 combined with decreased CD9 and CD20 expression [41], B-ALL/LBL associated with *KMT2A* rearrangements lacks expression of CD10 and CD24, but shows co-expression of myeloid markers CD15 and NG2 [42]. Identification of these phenotypic features by flow cytometry can provide the first clue to the presence of these significant genetic abnormalities.

Typically, blast cells in T-ALL/LBL express the following set of markers: CD45, CD7, cytoplasmic (cy) CD3, and nuclear TdT [36]. Additionally, variable expression of T-cell associated surface membrane antigens is seen: CD1a, CD2, CD3, CD4, CD5, CD8, CD99, TCRγδ, TCRαβ, dependent on the maturation stage of the blasts [36]. Although not included in the WHO-HAEM5, most flow cytometry laboratories routinely define T-ALL subtypes according to the European Group on Immunological Classification of Leukemia (EGIL): EGIL I–IV subtypes (Pro-T-ALL, Pre-T-ALL, Cortical T-ALL, Mature T-ALL) (Table 2) [37]. EGIL criteria provide additional information on the maturation stage of the lymphoblasts. In WHO-HAEM5, all of these subtypes fall into one entity – T-lymphoblastic leukaemia/lymphoma, NOS. The other entity – Early T-precursor lymphoblastic leukaemia/lymphoma (ETP-ALL/LBL) – is recognized by cyCD3^+^, CD7^+^, CD8^-^*,* CD1a^-^, CD5^lo^ (less than 75 % of blasts positive), and co-expression of one or more stem cell or myeloid markers (in ≥ 25 % of blasts) including CD13, CD33, CD34, CD117 or HLA-DR [37]. The major challenge in the identification of this entity by flow cytometry is the availability of a broad panel of monoclonal antibodies relevant to all hematopoietic cell lineages to efficiently distinguish ETP-ALL/LBL from acute undifferentiated leukemia and acute leukemia with T / myeloid phenotype. ETP-ALL/LBL might also be identified by RNA-seq, but for the routine diagnostic laboratory setting, flow cytometry seems more feasible, despite its limitations.

**Table 2:** Immunophenotypic criteria for the classification of T-cell lymphoblastic leukaemia/lymphoma (T-ALL/LBL) according to the European Group on Immunological Classification of Leukemia (EGIL)

	**Immunophenotypic markers**					
**Subtype**	*cyCD3*	*CD7*	*CD2*	*CD5*	*CD1a*	*smCD3*
Pro-T-ALL	+	+	–	–	–	–
Pre-T-ALL	+	+	+/-	+	–	–
Cortical T-ALL	+	+	+/-	+	+	+/-
Mature T-ALL	+	+	+/-	+	–	+

### Measurable residual disease (MRD) in ALL

MRD, the detection of low levels of residual leukemic cells, is important in risk stratification as a strong predictor of survival [4]. Several methods are used to quantify the level of MRD, including flow cytometry, RT-PCR or Next Generation Sequencing, each with unique advantages and disadvantages with respect to specificity, sensitivity, applicability and reproducibility [43]. They rely on the identification of a blast population specific target (e. g. immunoglobulin heavy chain (IGH)/T cell receptor (TCR) gene rearrangement, fusion transcript). The EuroFlow Consortium, the European Study Group on MRD detection in ALL (EuroMRD) and the I-BFM-FLOW-Network are dedicated to the standardization of protocols to assure accurate and consistent MRD detection for clinical application across different laboratories [44–46].

**Essential and desirable diagnostic criteria:**

In B-ALL, flow cytomorphology is essential to quantify more than 20 % of B-lymphoblasts with B-cell lineage markers. In B-LBL, blasts must show a B-cell immunophenotype, markers of immaturity and be immunoglobulin negative. The identification of specific recurrent genetic abnormalities is essential in B-ALL/LBL diagnosis.

An immunophenotypic profile associated with specific genetic alterations is desirable in both disease subtypes. For T-ALL, flow cytomorphology is essential for the detection of immature T-cells. Their presence outside of the thymus – in peripheral blood, bone marrow or other tissues – is strongly indicative of a precursor T-cell neoplasm. It is desirable to identify maturation stage of the blasts by flow cytomorphology and to discriminate between T-lymphoblastic leukaemia/lymphoma, NOS vs. Early T-precursor lymphoblastic leukaemia/lymphoma.

## Conclusion

In the new WHO-HAEM5, ALL and lymphoblastic lymphoma are taken together as lymphoblastic disease of B- and T-precursor cells. In B-ALL/LBL, new genetic subgroups have been defined, while in T-ALL/LBL, none have been identified, thus diagnosis is based on immunophenotyping and histology. For the first time, essential and desirable diagnostic criteria have been defined for ALL/LBL. The development and integration of many new genetic methods into routine diagnostics have greatly impacted the new classification. In the future, such new genetic methods will significantly impact further refinement of classification.
